# Ethical and social implications of public–private partnerships in the context of genomic/big health data collection

**DOI:** 10.1038/s41431-024-01608-9

**Published:** 2024-04-16

**Authors:** Ruth Horn, Jennifer Merchant, The UK-FR+GENE (Genetics and Ethics Network) Consortium, Ruth Horn, Jennifer Merchant, Mark Bale, Natalie Banner, Anne Cambon-Thomsen, Herve Chneiweiss, Angus Clarke, Yael Hashiloni-Dolev, Angeliki Kerasidou, Anneke Lucassen, Michael Parker, Christine Patch, Barbara Prainsack, Aviad Raz, Gesine Richter, Eva Winkler

**Affiliations:** 1 The Ethox Centre, Nuffield Department of Population Health, University of Oxford, Oxford, UK; 2 Institute for Ethics and History of Medicine in Society, Faculty of Medicine, University of Augsburg, Augsburg, Germany; 3 CNRS Law&Humanities/CERSA UMR-7109, University Paris-Panthéon-Assas, Paris, France; 4 Institut Universitaire de France, Paris, France; 5 University of Oxford, Oxford, UK; 6 University of Augsburg, Augsburg, Germany; 7 University Paris-Panthéon-Assas, Institut Universitaire de France, Paris, France; 8 Public Policy Projects Ltd, London, UK; 9 Genomics England, England, UK; 10 Inserm, University of Toulouse III, Toulouse, France; 11 CNRS, Inserm, Sorbonne University, Toulouse, France; 12 Cardiff University, Cardiff, UK; 13 Ben-Gurion University of the Negev, Be’er Sheva, Israel; 14 Wellcome Connecting Science, Oxford, UK; 15 University of Vienna, Vienna, Austria; 16 University of Kiel, Kiel, Germany; 17 University of Heidelberg, Heidelberg, Germany

**Keywords:** Medical ethics, Health policy

## Abstract

This paper reports on the findings of an international workshop organised by the UK-France+ Genomics and Ethics Network (UK-FR + GENE) in 2022. The focus of the workshop were the ethical and social issues raised by public-private partnerships in the context of large-scale genomics initiatives in France, Germany, the United Kingdom and Israel, i.e. collaborations where commercial entities are given access to publicly held genomic data. While the public sector relies on partnerships with commercial entities to exploit the full potential of the data it holds, such collaborations may have an impact on the return of benefits to the public sector and on public trust, and subsequently challenge the social contract. The first part of this paper explores the ways in which the four countries examined respond to the challenges posed to the social contract, and what safeguards they put in place to secure public trust. The second part presents three approaches to address the challenges of private-public partnerships in secondary data use. In conclusion, this paper offers a set of minimum requirements for these partnerships within solidarity-based publicly funded healthcare systems. These include the necessity of public-private partnerships to (1) contribute to the public benefit and minimise harm produced by the use of publicly held data; (2) avoid prioritisation of commercial interests over robust governance structures to guarantee benefits to the public and protect donors, especially marginalised groups; (3) side-step the pitfalls of the rhetoric of solidarity and be transparent about the challenges to return the benefits to ‘all’.

## Introduction

This paper is the result of the third workshop of the UK-FR+ Genomics and Ethics Network (UK-FR + GENE), a network that brings together academics, clinicians and policy-makers from France, Germany, the United Kingdom (UK) and Israel to reflect on ethical questions raised by genomic research and its implementation into clinical care [1]. Building on earlier workshops that explored the challenges that the collection, storage and sharing of genomic data may pose to the social contract (i.e. implicit or explicit agreements and expectations of the public towards science/medicine) in France, Germany and the UK [2], our third workshop investigated the very specific issue of public-private partnerships in genomic data collection, storage and transfer in these countries.

In the context of the increasing number of large-scale health data initiatives (e.g. Genomics England, Plan France Genomique 2025), public-private partnerships play a critical role in maximising the benefit of health data use (both primary and secondary uses) to enable the development of innovative treatments, public health surveillance and personalised healthcare. With the public sector in financial crisis across Western democracies, it has become apparent that the public sector cannot harness the full potential of the data it holds on its own and relies on partnerships with commercial entities. The involvement of such profit-oriented partners can, however, impact the return of benefits to the public sector and challenge public trust [3]. In this paper, by public-private partnerships, we refer to collaborations and agreements between public and private entities where private entities are given access to publicly held data.

Public opinion polls repeatedly demonstrate that citizens in democratic welfare states distrust profit-oriented companies accessing their data generated within the public healthcare system [4–6]. However, there might be some conditional public acceptance of these partnerships if regulations are in place and/or the aim is to develop health innovation [6, 7], and this has sometimes been described by some as ‘a necessary evil’ [8]. Various studies have also shown that the level of public distrust with regard to commercial entities accessing health data that is held by the public sector is higher in countries with a well-funded public healthcare system, compared to countries where the welfare state is perceived as less robust, unreliable and inefficient [9]. That is, the expectations of citizens towards the state to protect their personal data—and to use it for public and not merely commercial benefit—are higher in countries where public institutions have been responsible for promoting public benefit than in countries where citizens distrust the state in this respect [10].

As discussed in our last Consortium paper [2], the involvement of profit-driven companies and the increasing interest in turning health data into a wealth asset is challenging the social contract between a solidarity-based welfare state and its citizens; a contract that was established to ultimately benefit the public at large [3, 10, 11]. In this present paper we specifically explore, in the first part, the ways in which the countries we examined—the UK, France, Germany and Israel—attempt or fail to reinvigorate the social contract and secure trust from their citizens when entering for-profit partnerships relative to health data sharing for secondary use (i.e. for purposes other than the initial purposes of which the data were collected). In the second part, we present three approaches to addressing the challenges of the private-public partnerships in secondary data use. In conclusion, this paper will present what unites these three approaches, serving as a minimum requirement for these partnerships within solidarity-based healthcare systems.

## Part I: Practical approaches to and challenges of private-public partnerships

### United Kingdom: Developing trusted research environments

In recent years, the UK, whose healthcare service is the single holder of the biggest health dataset in the world, has emerged as a leader in the collection, storage and sharing of health data. One example, among others (e.g. Our Future Health, UK Biobank) is Genomics England (GEL) which was set up in 2013 to handle a range of genome-sequencing initiatives [2]. GEL is a company wholly owned by the UK government. It works with both public and private entities and has developed a secure research environment to protect donor data from abusive secondary use. All access to the data base known as the National Genomic Research Library (NGRL), is regulated, operating (metaphorically) as a ‘reading’, not a ‘lending library’. The platform allows approved private and public researchers to access—but not extract—samples, genomic data and other associated health data that have been de-identified. Access is given only when the proposed research aims to find new treatments, improve analysis of large datasets, share knowledge among researchers and clinicians to advance research, develop novel drugs and diagnostics, propose clinical trials, and lead to other pertinent research. There is a list of unacceptable uses and commercial sectors that will be denied access [12]. This access requirement demonstrates how GEL strives to ensure that the data it manages is used to promote public benefit and foster the social contract [1].

However, several challenges remain where access to publicly held data is given to commercial companies. This is particularly the case in exploratory early-stage research where potential public benefits for patients or healthcare systems cannot be clearly determined, yet companies may overpromise the likelihood of public benefits to access the data. Finally, in order to retain a competitive advantage, commercial entities are willing to embrace only limited openness about their aims and intentions, all of which have an impact on public trust and trustworthiness.

Even though GEL’s research environment aims to address these challenges, much remains to be accomplished. There is a risk of complacency if the use of a secure data environment is thought to solve all public questions and concerns. In fact, the monitoring of public as well as commercial research and its outputs in practice remains difficult and resource intensive, raising itself ethical questions. Although there are good reasons to prioritise monitoring, the benefits need to be weighed against the financial costs (funds that could be spent on research or other benefits) and the limitations on research when some aspects of scientific practice become impossible or more difficult, due for example to restrictive data sharing policies.

### France: concerns about overseas commercial companies

The Plan France Médecine Génomique 2025 was launched in 2015 to drive the development of genomic medicine by sequencing 235,000 genomes per year between 2020 and 2025. This data, among other health data, is held by the French Health Data Hub (FHDH) [13], setup in 2019, to guarantee ‘a transparent, simplified and unified access to health data to improve the quality of care and patient support’ [14]. It includes 56 public and private stakeholders and is mainly, even if not solely, publicly funded. The FHDH serves as a unique entry point facilitating access to health data for research, and its aim is to contribute ‘to public interest’, respect patient rights and ensure transparency with civil society [15]. The platform’s objective is to offer, among others, a high level of security, storage and data analysis capacities.

The FHDH held data, will be accessible to project coordinators—both in the public and private sector—where their research contributes to the ‘public interest’. Applications to access data will be reviewed and approved by an independent committee and the National Commission for Data Protection and Liberties (CNIL). Like the NGRL, data can only be remotely accessed and processed on the FHDH platform without any possibility of downloading it. Again, we observe here a concerted effort by the government to respect what the social contract entails with emphasis on the importance of the ‘public interest’, a concept that is akin to the concept of ‘common good’ [16].

However, a recent case, as yet unresolved, demonstrates the interpretation of what constitutes public good or public interest can be complicated and controversial on the ground. The US-based company Microsoft and the French government signed an agreement to store the entirety of the FHDH health data on Microsoft’s Azure’s cloud that was deemed at the publics’ interest. As Lombrail et al. point out, this raises at least three major concerns: (1) the influence of financial considerations on the FHDH and how value is created that could be contrary to scientific integrity and the public interest, as well as questions about the long term FHDH economic model; (2) the fact that this data comes under US legal jurisdiction and what this means for data privacy and protection as required by the GDPR; (3) the existence of a centralised cloud that is vulnerable to hacking [17].

Following intense criticism by the CNIL, a provision was inserted to this contract with Microsoft that allows any French citizen who has become aware of illicit use of their data to sue the company. Here, the state is fulfilling a minimum requirement of protection of its citizens yet leaving citizens alone in addressing the issue.

### Germany: the predominance of the precautionary principle to not share data with profit-oriented companies

In Germany, up until recently, health data was scattered and stored across the country in various hospitals and research centres governed by each federal state’s own data protection policy. This is due to one of the main differences between Germany and the UK’s NHS: there is no national central healthcare system. Instead, there are different providers that operate the hospitals (federal states, municipalities, churches, etc.). In 2018, the German Medical Informatics Initiative was set up to start a process of building infrastructures for the linking and sharing of clinical data from (mainly) public university hospitals [13]. Its aim is to develop a structure that produces research findings for the direct benefit of patients [18].

For a long time, pharmaceutical and other commercial entities who wanted access to anonymised patient data, pointed out that Germany lagged behind in the digitalisation of health data, placing the country on rank 16, second to last, in an international comparison of the development of digital health strategies [19]. The Digital Healthcare Act of 2019, passed in an effort to reduce delays in the process of digitalisation in Germany, does not allow commercial companies to access health data held by public institutions and focuses on data sharing among public institutions across the federal state [20]. An important development with regard to genomic data sharing is the National Strategy for Genomic Medicine, GenomDE, whose model 1 regulation will come into effect in 2024 to allow for comprehensive genome sequencing in the context of a structured clinical treatment workflow in rare and oncological diseases and the integration of clinical and genomic data. The infrastructure facilitates the analysis of the acquired data to improve medical care.

According to the data strategy plan of the German federal government, regulation of data handling and sharing ought to go hand in hand with the protection of citizens’ ‘general right of personality, consideration of their private life and their informational self-determination (…) and protection against discrimination’ [21]. The strong emphasis on the protection of privacy and confidentiality might also refer to the historical experience with two political regimes that used their control over private information against their own citizens, and, particularly, against opponents of the regimes [22]. Today, to warrant citizens’ trust, the government is making an effort to ensure that public concerns are taken into account and strict governance is in place as digitalisation is slowly implemented [23].

### Israel: the unreliable state versus unfair but professional commercial companies

In Israel, the Maccabi Health Services (MHS), the second largest health fund, has established the first and only large-scale Israeli population-based DNA biobank.

In 2018, a new, national DNA biobank (called ‘Psifas’, mosaic, in Hebrew) was launched by the government, as part of Israel’s Digital Health Initiative. According to The Genetic Information Act (2000), DNA data may be shared for purposes of legally approved research, or publication in a scientific journal, on condition that (1) the genetic information is transmitted without any identifying detail; or (2) the individual data subject has consented in writing to the transmission of genetic information. International data sharing (including with commercial companies) is permitted only for research purposes, subject to the approval of the ‘Supreme Helsinki Committee’, in accordance with the provisions of the Privacy Protection Regulations (Transfer of Information to Databases Outside the State’s Boundaries)—2001.

A recent interview study showed that even though DNA donors in Israel did not trust the state to reliably monitor the legitimate uses of their data and would apply any knowledge gained directly for the common good, they were nevertheless willing to donate their data [24]. However, in that study respondents were all Jewish and the sample size was rather small; future studies should include individuals from the diversity of ethnicities in the Israeli population, which Psifas aims to include samples from. People considered that in a digital age, where individual data is routinely collected by social media and other commercial companies, it has become impossible to keep control over one’s own data. The authors of the study found that this reservation towards the state and HMOs (Health Maintenance Organisations) did not significantly differ from the attitude toward commercial for-profit companies that operate within the medical field. Yet, while the interviewees perceived the state as unreliable and inefficient, commercial companies were perceived as unfair but at least professional. Many of the interviewees who confirmed their willingness to donate their data despite their distrust in governance frameworks, explained that they believe it is impossible to protect their data, yet they would at least ‘hope’ their data would achieve something good and contribute to improving the health of others. By explaining how they donate ‘with eyes shut’, the participants (many of whom were approached by biobank representatives while waiting in line at HMO clinics) expressed their ambivalent motivation to donate data.

The findings of this study challenge the observed relationship between public trust (in institutions and in the benefit of data-based medicine) and the willingness of citizens to donate data as well as the emphasis on solidarity as a motivation to donate data. The authors describe the eyes-shut-strategy as one where participants donate half-heartedly while preferring not to think about what happens with their data and ‘hoping’ that something good will come out of it. Even though people could come to donate data without trusting, from an ethical perspective, it seems more desirable to build and warrant public trust in data donation through effective communication about the value of data, i.e. the benefits gained from it as well as about existing governance structures, particularly when private companies enter the field [25].

## Part II: three approaches to address the challenges of private-public partnerships

As was discussed in the workshop and through our comparative observations above, the four countries studied present national context-specific issues that in turn result in different concerns depending on the country. Was it therefore possible to conceive of a general model that could address all these specific concerns? Several proposals were advanced.

### A solidarity grounded partnership model

Considering the challenges posed by partnerships between solidarity-based healthcare systems and commercial companies and, in particular, their impact on public trust, we first discussed the possibility of a solidarity grounded partnership model [1].

Starting from the premise that solidarity implies ‘responsibility […] togetherness and commitment to the common good’ [26], it is the foundational principle of publicly funded healthcare systems who, by their very nature, are committed to provide the best possible care for all according to their needs and promote a sense of mutual responsibility based on contribution by all according to their means. These characteristics contribute to the development and establishment of public trust [27] because citizens can reasonably believe that public institutions will serve the common good and represent collective interests [28]. Yet, as public healthcare systems enter partnerships with profit-oriented commercial companies, the trust in public institutions maintaining their solidaristic character and remaining entirely committed to the common good has the potential to come under significant pressure.

Against this background, to build trustworthy partnerships between public and private institutions that respect both the interests of profit-oriented private companies and those of public institutions, this approach would suggest that these partnerships should be built on norms of solidarity and public benefit and put the public’s expectations and concerns centre stage. In the context of public healthcare systems giving commercial entities access to their data, this would require: (1) preferential access of public healthcare systems to goods and services developed using their data; (2) using data only where this can be expected to improve health and healthcare and not solely to serve the interests of private insurance companies or other commercial interests; (3) transparency about conflicts of interests, and how they are managed and resolved; (4) a monitored data-visiting model of access similar to the one implemented by Genomics England and the FHDH, rather than a data-sharing model where the data leaves its initial environment [3].

### A data-solidarity approach to public-private partnerships

While the above model focuses on the nature of public-private partnerships, another approach to solving challenges of these partnerships puts emphasis on a solidarity-based data governance framework [11]. Taking its inspiration from the basic tenets of solidarity-based healthcare systems and applying them to digitalised information, ‘data solidarity’ emphasises collective control, responsibility and oversight. It is argued that this has become urgent, as attempts to increase the control of individuals over the use of their own data no longer suffice within contemporary political and economic realities. Hence, data solidarity necessitates that the benefits and the risks of digital practices needs to be borne by societies collectively and shared equitably.

How is this to be achieved? From a general standpoint, regulation should be designed for specific types of data use, based on the benefits and harms that such data use is likely to produce [29]. More precisely, a threshold of acceptable risk of data use needs to be defined and any risks to individuals or communities beyond this threshold should be either mitigated [30] or, where this is not possible, firmly outlawed, with fines and robust enforcement mechanisms that deter even large corporations. Indeed, these considerations all point to the fact that reliance on self-regulation by the corporate sector is futile.

Data use in the context of public-private partnerships that is likely to create significant public benefits without posing unacceptable risks should receive more public support than is currently the case—by easing regulatory burdens where possible and appropriate, or by providing financial and other support. In cases where data use does not create significant public value but yields commercial profits, some of these profits should be returned to the public domain. This however presumes that the public benefit gained through commercial profits clearly outweighs any possible risks, and that the profits are distributed appropriately according to people’s need. Indeed, large companies using data that has been collected within the public healthcare sector should share profits with the public, for example, through fair taxation, or via additional benefit sharing measures at the national level or with specific communities. It is also important that individual people should not be paid for their data, as this would aggravate social and economic inequalities wherein the poor pay with their data for services and goods they cannot afford [31]. Instead, data should be treated as collective property that is to be governed democratically—either at the national, regional or even local commons level. This approach prevents quasi-monopolist commercial companies or those that have broken legal or ethical rules to be excluded from data use or to have restrictions imposed on their use. Last but not least, this model would require that in situations where data harms were to emerge without any law having been broken or without anybody being legally liable (which means that those who experienced the harm have no access to legal remedies), there should be support for the person(s) harmed independent of their social or economic status. Likewise, whistle-blower protection in the context of data use should be strengthened, such as presented by the European Data Protection Supervisor guidelines in December 2019 [32].

### A rights-based approach to public-private partnerships

A third approach to addressing the challenges posed by public-private partnerships aims to provide a normative framework that includes all relevant stakeholder perspectives on the use of data generated in the public health sector by profit-oriented companies: patients/data subjects, private companies, the public, physicians and public healthcare institutions [18]. This model takes a rights-based approach [33] according to which all persons have both rights (e.g. right to privacy, informational self-determination, right to freedom of research) and certain duties (e.g. respecting transparency, accountability and liability, participation and representation in decision processes). This account recognises individual rights as a core element of liberal democracies. It also addresses the question of the moral status of companies and opts in favour of an account that ascribes moral rights, duties and responsibilities to companies [34]. While this approach recognises companies’ fundamental right to research and to pursue profit, this does not mean that companies have a right to access patient data from the public healthcare system. Rather, the main reason why companies should have access to patient data is public benefit. It is therefore important to reconcile the legitimate interests and rights of individual stakeholders as best as possible and to mitigate tensions between them through concrete measures.

On this model, one of the conditions for companies to have access to patient data would be that there is also a contribution to the public benefit, and that this benefit outweighs any risks, is accessible and distributed appropriately depending on people’s needs. Examples where public benefit would not be achieved is when the ensuing company product does not convey any real added value, convey risks that outweigh benefits, is overpriced or is not available on the domestic market at all. Therefore, private entities must agree to limits on their pursuit of profits and accept governance frameworks set by regulators.

This implies that private companies: (1) use (access to) the data solely for research that aims to improve health or the healthcare system; (2) prove that their products supported by public data benefit patients and the public healthcare system; (3) respect the principles of accountability and transparency; (4) may seek profit after approval but have a fair price that is not prohibitive or unaffordable for the public health care system; (5) publicly register their research protocols and in determining fair-pricing reveal to what extent they relied on patient data; (6) are held accountable for data security, protecting patients’ privacy and minimising risks; (7) must include patient representation and participation in the establishment of data use frameworks to respect their interests.

### Discussions and questions emerging from the workshop

Many issues were raised in the discussion portion of the workshop, the most acute one being around the notions of social contract and solidarity. It was initially suggested that solidarity forms the basis of a social contract between citizens and public institutions such as public healthcare. However, it was also argued that the concept of the social contract is separate from the concept of solidarity and that solidarity is not the necessary or sole foundation that illustrates the shortcomings and problems of public-private relationships. This suggests that it may be more useful to observe the existence of a range of different but largely complementary ways in which solidarity could be an organising principle of a health system.

As outlined above, participants argued that there are other foundations or arguments that could be candidates for explaining and counterbalancing the challenges of public-private relationships. One of these was the above-mentioned rights-based approach. With respect to this approach, and also to some degree to the other approaches, questions were raised about whether and to what extent the requirement to engage private companies in contributing to the public benefit could be achieved in practice as this goes against the competitive edge of private companies. Indeed, it was argued that private companies will by nature seek the maximisation of their profits, even in the event that they might be willing to pay an initial price for their ensuing benefits.

Further to the rights-based approach, debate ensued over what entity would decide how these rights are protected and conflicting interests ought to be balanced. It was argued that it would need to be a public organisation as the representative of democratically defined rights and responsibilities. However, it was conjectured that private companies might find fault with that and challenge these rights and responsibilities in a public-private relationship. Indeed, the question was raised of why a public entity should have the upper hand in this definition and distribution and lay-stakeholders such as patient and participant representatives were discussed as important agents in overlooking this process. Finally, it was acknowledged that oversight also requires resources to be available which could raise issues for countries that are unable to afford e.g. secure data hubs in the form of research ‘libraries’.

Subsequent to the rights-based approach debate, discussions focused on the broader notion of solidarity following the presentation on data solidarity.

In particular, there was a debate on whether citizens have a duty to donate their data and whether this could even be based on the concept of solidarity [35]; an obligation that is not defended by the data solidarity approach [11]. It was discussed that there might also be other reasons, such as the duty to act in the public interest and duty of assistance, to justify such a moral obligation [36]. It was questioned whether such a duty could be required without ensuring upstream that results from data-based research will favour justice and ‘enhance human flourishing’ [37]. Discussion was also devoted to the notion that individuals could choose not to be reluctant to donate data despite the general mistrust, because in fact it could become beneficial to them at term. At the same time, it was suggested that a more in-depth discussion is required, notably a reflection on the issue of free-riding, meaning citizens benefiting from other patients’ data without giving their own [38]. Furthermore, regarding the claim made by the data solidarity approach, that individuals should not be paid for their data to avoid exploitation of poor people, critique was expressed that this could be patronising and that there are good ethical reasons to reasonably reimburse data donators.

Finally, it was discussed to what extent the concept of solidarity can even be employed as long as not all groups of society are represented in the datasets and data diversity is far from being achieved [39]. That said, making visible the invisible (marginalised groups) is not beneficial in all contexts, because robust safeguards are often not in place for these groups and the potential exists for their data to be used to their disadvantage. Generally, weighing the risks and harms regarding the secondary use of data remains difficult but, as a process subject to ongoing debate, such an objective could ultimately be achieved. We also need to be sensitive to the rhetorical use of ‘solidarity’ to impose obligations on ‘all of us’ to donate data [40], as this may mask the fact that the benefits for ‘all’ are uncertain and curtailed by commercial interests and even have the potential to exacerbate pre-existing inequities. With new technological advancements, increased awareness of past transgressions and a recognition that extractive research causes harm in a variety of ways, we hope that communication efforts can increasingly focus on genuine engagement in genomic research, especially when it comes to underrepresented populations [41].

## Conclusion

Informed by the discussion about the approaches outlined above, the workshop participants reached consensus on three conditions that should apply to public-private partnerships in the use of secondary data.

First, participants observed that, to maximise the benefit of data use to the public, engagement in public-private partnerships is unavoidable.

Second, it was thought that these partnerships should contribute to the public benefit in the realm of healthcare, even though the means for this- and its evaluation-are still open to discussion. Different regulations should apply depending on the benefits or harms the use of data is likely to produce. While access to data should be facilitated where its use provides clear public benefit, any use that poses risks to individuals or communities should be prohibited. Such risks include profit-making from data commerce unbeknown to the donor, illegal transfer of data to law enforcement and/or other government institutions, etc. Hence, at this stage, allowing any risk to individuals or communities in the framework of public-private partnerships was adamantly rejected.

Third, participants also agreed on the inherent dangers that must be avoided in these partnerships, such as prioritising commercial interests over sufficient oversight and the generation of public benefit. With regards to this oversight, the participants concluded that there must be a robust and trustworthy governance structure in place that guarantees a return to the public good and protects donors, especially marginalised groups. Conflicts of interests and commercial interests must be made transparent and research protocols must clearly outline how benefits will be returned to the public and the public sector (e.g. preferential access to goods and services developed). Although these three conditions leave room for interpretation in their practical implementation, they are a first step towards establishing sustainable public trust and confidence in the oversight of the use of health data in the interests of all.

Finally, it is important to remind ourselves that the notion of solidarity can be particularistic (national, ethnic, or religious solidarity). As a result, rather than dismissing the option of solidarity, it is important to understand why it has become a point of contention, especially when it comes to underrepresented populations. In particular, future endeavours will need to address solidarity and benefit-sharing not as contradictory but as inter-connected.
